# Evaluation of the health-related quality of life and associated factors in Zimbabwean adults living with HIV: a cross-sectional study

**DOI:** 10.1186/s13104-023-06536-3

**Published:** 2023-10-04

**Authors:** Tendai Orial Tigirigi, Grace Yolanda Sithole, Princess Chakara, Gracious Z. Chirombo, Annamore Rutendo Chiweza, Trish R. Mubayiwa, Sidney Muchemwa, Dixon Chibanda, Jermaine M. Dambi

**Affiliations:** 1 Rehabilitation Sciences Unit - Faculty of Medicine and Health Sciences, University of Zimbabwe, PO BOX AV 178, Avondale, Harare, Zimbabwe; 2 Mental Health Unit - Faculty of Medicine and Health Sciences, University of Zimbabwe, PO Box A178, Avondale, Harare, Zimbabwe

**Keywords:** Common Mental Disorders: HIV/AIDS, HRQoL, Zimbabwe

## Abstract

**Objective:**

HIV remains a global burden, with the Sub-Saharan Africa (SSA) region reporting the largest number of people living with HIV/AIDS (PLHIV). An exponential improvement in the accessibility and uptake of antiretroviral treatment across SSA has significantly improved outcomes for PLHIV. Hence, HIV care goals have shifted from reducing mortality and morbidity to improving health-related quality of life (HRQoL). This study uses generic and condition-specific HRQoL outcomes to holistically determine the HRQoL of Zimbabwean adult PLHIV and associated factors. HRQoL is a dynamic subject construct that warrants continuous evaluation to provide meaningful feedback to various stakeholders. We enrolled 536 adult PLHIV in Zimbabwe. Collected data were analyzed through descriptive statistics and multivariate binary logistic regression.

**Results:**

Our study shows a high HRQoL perception by Zimbabwean PLHIV. Anxiety, depression, and poor environmental health were widely reported domains influencing HRQoL. Also, being aware of HIV status for over a year, not experiencing an adverse event, being married, having adequate finances and food security and having higher educational status were associated with higher HRQoL. It is essential to integrate mental health care into routine HIV care to improve treatment outcomes and HRQoL. Last, implementing bespoke multisectoral HRQoL-enhancement interventions is paramount.

**Supplementary Information:**

The online version contains supplementary material available at 10.1186/s13104-023-06536-3.

## Background

HIV remains a global burden, with the Sub-Saharan Africa (SSA) region reporting the largest number of people living with HIV/AIDS (PLHIV) [1, 2]. Encouragingly, the SSA region has made considerable strides in HIV care, ranging from HIV prevention campaigns to scaling up antiretroviral treatment (ART) [2, 3]. Consequently, there has been an exponential improvement in the accessibility and uptake of ART in primary health settings across the SSA, with resultant significantly improved outcomes for PLHIV [4]. For instance, in 2023, over 95% of Zimbabwean PLHIV attained viral load suppression joining four other countries in Eastern and Southern Africa that have met the UNAIDS 95-95-95 targets [5].In addition, Zimbabwe’s repeal of Sect. 79 in the Criminal Law Code, which criminalized HIV transmission, improves access to HIV care and treatment [6]. However, the World Health Organisation (WHO) acknowledges that viral suppression is not the ultimate care/treatment goal [7]. HIV care goals have shifted from reducing mortality and morbidity to improving health-related quality of life (HRQoL) [7, 8]. HRQoL measures the extent of perceived individual functioning in multiple domains, i.e. physical, social and mental health over time [9, 10]. Evaluation of HRQoL is pivotal to patient-centred HIV care, where therapeutic goals include preventing disease progression, reducing symptom complications, improving skills and modifying the severe psychosocial consequences associated with the disease [8]. Moreover, HRQoL measures can be incorporated into models that inform the economic evaluation of HIV/AIDS treatment services, making them pivotal in resource allocation decisions and policy formulations [4, 11, 12].

It is thus essential to understand the HRQoL and its determinants in PLHIV to develop bespoke interventions. Systematic reviews have shown that age, treatment adherence, depression, social support, employment, and HIV-related stigma are associated with HRQoL in PLHIV [11, 13, 14]. However, methodological differences, including heterogeneity of outcome measures across studies, limit external validity. For instance, generic and disease-specific tools have been previously used in isolation or combination to measure HRQoL in PLHIV [15, 16]. Generic instruments can provide results comparable to the general population and other conditions [17]. Conversely, HIV-specific HRQoL outcomes have expanded content validity and more nuanced psychometric performance in measuring HRQoL in PLHIV [18]. However, few studies have applied generic and condition-specific outcomes to holistically understand the HRQoL of PLHIV, particularly in low-income countries where the HIV burden is enormous [19]. This study used generic and condition-specific HRQoL outcomes to determine the HRQoL of Zimbabwean adult PLHIV and associated factors. HRQoL is a dynamic, subjective construct that warrants continuous evaluation to provide meaningful feedback to various stakeholders (e.g., patients, clinicians, and policymakers). A previous study from a single urban facility in Zimbabwe (n = 257) found high HRQoL among PLHIV in 2015. Even so, Zimbabwe has undergone a massive economic decline over the decades, negatively impacting the HRQoL of both PLHIV and the general population. For example, hyperinflation and chronic public health financing have negatively impacted the public healthcare system. More importantly, COVID-19-induced disruptions to livelihoods and HIV care have impacted the HRQoL of PLHIV, hence the need for this study to inform evidence-based care.

## Methods

### Study design

Cross-sectional study.

### Setting

This study was done at three tertiary-level institutions and six polyclinics (primary healthcare facilities) in Harare, the capital city of Zimbabwe. All study sites have dedicated HIV testing, and treatment facilities, including opportunistic infections, care and sexual and reproductive health services.

### Participants

We recruited adult PLHIV ≥ 18 years who were willing and able to provide written consent and could understand either English or Shona (a Zimbabwean native language). Exclusion criteria included mentally unstable adults living with HIV and acutely unwell patients requiring emergency care.

### Sampling and sample size calculation

We assumed an equitable HRQoL of PLHIV from a South African sample yielding a mean overall HRQoL of 12.57 (SD 4.65) (µ_0_) [20]. Our minimum sample size was 525 at β = 0.8, 95% CI and assumed mean overall HRQoL of 12.0 (µ_1_). We determined our sample size using STATISTICA Version 14. Participants were recruited using a consecutive sampling method.

### Instrumentation

We used the WHOQOL-HIV-BREF [21] and Eq. 5D-5 L [22] for disease-specific and generic HRQoL evaluations, respectively. The WHOQOL-HIV-BREF consists of 31 items covering six domains, that is spirituality, environmental health, physical health, psychological health, level of independence, social relationship, religion, and personal beliefs. The scores range from 4 to 20: higher scores suggest better HRQoL [21]. The Eq. 5D-5 L comprises five dimensions, i.e., mobility, self-care, usual activities, pain/discomfort, and anxiety/depression [22]. Scores across the five domains are transformed into a summative utility score. Both outcomes are available in Shona, a Zimbabwean native language and are validated. Participants also completed a socio-demographic questionnaire consisting of variables such as gender, age, employment status and level of education (See Supplementary File 1).

### Procedure/ethical considerations

We collected data in April 2022 using the Kobo Collect application installed on Android tablets. We embedded mandatory field codes in the electronic data collection tool to negate missing data. Participants signed the consent forms and were interviewed in a private room.

### Data analysis

Descriptive statistics (e.g., means and frequencies) were used to describe participants’ characteristics and responses to the standardised outcome measure Bivariate and multivariate binary logistic regression were used to evaluate factors associated with HRQoL. All tests were done at α = 0.05 using SPSS 27.

## Results

### Participant characteristics

Most participants were females (60.6%), completed secondary education (75.2%), knew their HIV status for more than a year (86.6%), and had disclosed their HIV status (80.0%). Those on ART (97.8%) had been on treatment for more than a year (84.1%) and were always adherent to ART (89.0%). Further, most participants were non-smokers (91.2%), non-alcohol consumers (74.6%), did not use substances (96.3%), and had not experienced any adverse events in the past month prior to data collection (71.6%) - Table 1.

**Table 1:** Participant characteristics, N = 536

Variable	Attribute	Frequency, n (%)
Gender	Female	325 (60.6)
	Male	211 (39.4)
Highest Level of Education	None	5 (0.9)
	Primary	43 (8.0)
	Secondary	403 (75.2)
	Tertiary	85 (15.9)
Marital/Relationship status	In a relationship	123 (22.9)
	Currently married	155 (28.9)
	Widowed	54 (10.1)
	Not in a relationship	158 (29.5)
	Other	46 (8.6)
Employment Status	Unemployed	130 (24.3)
	Informally employed	127 (23.7)
	Formally employed	103 (19.2)
	Student	88 (16.4)
	Housewife	53 (9.9)
	Other	35 (6.5)
Duration since HIV diagnosis	Days	7 (1.3)
	Weeks (less than a month)	7 (1.3)
	Months (less than a year)	26 (4.9)
	1 year	32 (6.0)
	More than 1 year	464 (86.6)
Mode of HIV infection	Perinatally	221 (41.2)
	Sex	272 (50.7)
	Injecting drugs	7 (1.3)
	Blood products	19 (3.5)
	Other	21 (3.9)
ART intake status	Yes	524 (97.8)
	No	12 (2.2)
Duration on ART	Days	4 (0.7)
	Weeks (less than a month)	5 (0.9)
	Months (less than a year)	29 (5.4)
	1 year	35 (6.5)
	More than 1 year	451 (84.1)
ART adherence	Not at all	6 (1.1)
	Sometimes	39 (7.3)
	Always	477 (90.0)
Smoking status	Yes	47 (8.8)
	No	489 (91.2)
Alcohol intake	Yes	136 (25.4)
	No	400 (74.6)
Drugs/substances intake	Yes	20 (3.7)
	No	516 (96.3)
HIV disclosure	Yes	429 (80.0)
	No	107 (20.0)
Hospital admission in the past year	Yes	57 (10.6)
	No	479 (89.4)
Adverse events in the past month	Yes	152 (28.4)
	No	384 (71.6)

### Correlations between main outcome variables

WHOQOLHIV-BREF scores were positively and highly correlated to EQ-5D 5 L utility scores (r = .549, p < .001).

### Factors associated with HRQoL

#### Binary logistic regression

In bivariate analysis, low educational attainment, being widowed/divorced, unemployment, low financial status, food insecurity, previous hospital admission, being diagnosed with HIV for less than a year, and experiencing adverse events in the past month were significantly associated with reduced HRQoL - Table 2.

**Table 2:** Factors associated with HRQoL – Unadjusted odds ratios

		WHO-QOL BREF		EQ-5D Utility	
Variable	Attribute	COR (CI)	p-value	COR (CI)	p-value
Gender	Female	1.39 (0.98- 1.97)	*0.067*	1.23 (0.85:1.78)	0.266
	Males (Ref)	1		1	
Age		0.99 (0.98: 1.01)	0.209	0.99 (0.97: 1.00)	*0.042*
Education	Primary	0.35 (0.17: 0.72)	*0.005*	0.28 (0.13: 0.59)	*< 0.001*
	Secondary	0.49 (0.30: 0.80)	*0.005*	0.54 (0.31: 0.93)	*< 0.001*
	Tertiary (ref)	1		1	
Relationship status	Not in a relationship (Ref)	1		1	
	In a relationship	0.96 (0.60: 1.53)	0.855	1.16 (0.69: 1.94)	0.579
	Currently married	1.09 (0.69: 1.70)	0.721	0.87 (0.54: 1.40)	0.574
	Widowed/Divorced	0.56 (0.34: 0.94)	*0.028*	0.43 (0.256: 0.73)	*0.002*
Employment status	Unemployed	0.59 (0.41: 0.83)	*0.003*	0.70 (0.48: 1.01)	*0.054*
	Employed (Ref)	1		1	
Financial adequacy	Adequate (Ref)	1		1	
	Inadequate	0.35 (0.24: 0.49)	*< 0.001*	0.34 (0.23: 0.50)	*< 0.001*
Food security	Adequate (Ref)	1		1	
	Inadequate	0.23 (0.15: 0.34)	*< 0.001*	0.35 (0.24: 0.52)	*< 0.001*
Hospital admission status	Yes (ref)	1		1	
	No	2.22 (1.25: 3.95)	*0.007*	4.84 (2.68: 8.78)	*< 0.001*
Adverse events	Yes (ref)	1		1	
	No	2.4 (1.63: 3.54)	*< 0.001*	3.06 (2.07: 4.53)	*< 0.001*
Smoking status	Yes (Ref)	1		1	
	No	1.53 (0.83: 2.82)	*0.17*	1.69 (0.92: 3.11)	*0.092*
Alcohol intake	Yes (Ref)	1		1	
	No	1.01 (0.68: 1.50)	0.958	1.25 (0.83: 1.87)	0.286
Drug substances intake	Yes (Ref)	1		1	
	No	2.54 (0.95: 6.79)	*0.063*	1.41 (0.56: 3.58)	0.466
HIV awareness	< one year				
	≥ one year (Ref)	0.32 (0.16: 0.66)	*0.002*	0.47 (0.24: 0.90)	*0.023*
HIV cause	Congenital	1.6 (0.84: 3.17)	*0.148*	2.50 (1.26: 4.92)	*0.008*
	Sex	1.05 (0.54: 2.00)	0.895	1.23 (0.64: 2.36)	0.539
	Other (Ref)	1		1	
Disclosure status	No	0.96 (0.63: 1.47)	0.844	1.81 (1.12: 2.94)	*0.016*
	Yes (Ref)	1		1	
ART intake status	Yes (Ref)	1		1	
	No	0.62 (0.20: 1.99)	0.423	1.58 (0.42: 5.91)	0.497
Art intake duration	Less than a year	0.49 (0.26: 0.88)	*0.017*	0.61 (0.34: 1.10)	*0.103*
	More than a year (Ref)				
ART adherence	Yes (Ref)	0.43 (0.08: 2.38)	0.334	0.52 (0.10: 2.61)	0.427
	No	1		1	

*Note*: Italic value highlights a statistically significant association between variables in context

#### Multivariate logistic regression analysis

After adjustment for co-variance, not experiencing an adverse event, being aware of HIV status for more than a year, being currently married, having adequate finances and food security and having higher educational status were associated with higher HRQoL - Table 3.

**Table 3:** Factors associated with HRQoL – Adjusted odds ratios

		B	S.E.	Wald	df	Sig.	Exp(B)	EXP(B) 95% C.I.	
								*Lower*	*Upper*
WHOBREF	Did not experience an adverse event	0.70	0.22	10.35	1	*0.001*	2.01	1.31	3.07
	HIV awareness- less than a year	-1.25	0.40	9.70	1	*0.002*	0.29	0.13	0.63
	Relationship status- Not in a relationship (Ref)			9.54	3	*0.023*			
	In a relationship	0.12	0.26	0.20	1	0.656	1.13	0.67	1.89
	Currently married	0.61	0.26	5.30	1	*0.021*	1.84	1.09	3.08
	Widowed/Divorced	-0.24	0.29	0.66	1	0.416	0.79	0.44	1.40
	Financial inadequacy	-0.82	0.21	15.46	1	*0.000*	0.44	0.29	0.66
	Food insecurity	-1.29	0.23	30.95	1	*0.000*	0.28	0.18	0.43
	Constant	0.38	0.25	2.28	1	0.131	1.46		
EQ-5D	No past hospital admissions	1.49	0.34	19.4	1	*0.000*	4.44	2.29	8.63
Utility	Did not experience an adverse event	0.95	0.23	17.8	1	*0.000*	2.59	1.66	4.02
	Relationship status - Not in a relationship (Ref)			11.1	3	*0.011*			
	In a relationship	0.31	0.29	1.2	1	0.283	1.37	0.77	2.43
	Currently married	0.11	0.28	0.1	1	0.701	1.11	0.65	1.91
	Widowed/Divorced	-0.69	0.30	5.3	1	*0.022*	0.50	0.28	0.91
	Financial inadequacy	-0.82	0.23	13.0	1	*0.000*	0.44	0.28	0.69
	Food insecurity	-0.68	0.23	8.8	1	*0.003*	0.51	0.32	0.79
	Lower educational status	-0.73	0.34	4.5	1	*0.034*	0.48	0.25	0.95
	Constant	-0.52	0.37	2.0	1	0.161	0.59		

*Note*: Italic value highlights a statistically significant association between variables in context

## Discussion

This study evaluated the HRQoL of Zimbabwean PLHIV and associated factors. Overall, our study shows a high HRQoL for PLHIV in Zimbabwe. Anxiety, depression, and poor environmental health were widely reported domains influencing HRQoL. Also, being aware of HIV status for over a year, not experiencing an adverse event, being currently married, having adequate finances and food security and having higher educational status were associated with higher HRQoL. Study participants expressed a higher HRQoL evaluation despite the ongoing socio-economic challenges. These findings are comparable with previous studies conducted in Zimbabwe [19], Zambia and South Africa [23, 24], which yielded these mean EQ-5D VAS utility scores; 0.81 ± 0.30 and 0.88 ± 0.10 and 0.89 ± 0.03, respectively. A high perception of HRQoL could reflect cultural conceptualisation or a manifestation of resilience in PLHIV. Most of the study participants had lived with HIV for over a year and may have developed resilience in living with a chronic condition. Systematic reviews have shown that resilience progressively develops and is associated with improved positive well-being, treatment adherence, and posttraumatic growth in PLHIV [25, 26].

Despite the high HRQoL perception/evaluation, most study participants reported problems in the anxiety/depression dimension and poor environmental health. Systematic reviews and meta-analyses show that depression is two to three times more common in PLHIV than in the general population [27]. Risk factors for poor mental health functioning in PLHIV include challenges coping with a recent HIV diagnosis, adverse external environmental factors (e.g., stigmatisation, poor living conditions), the experience of living with a chronic illness and personal/marital relationships challenges [28–31]. Therefore, it is essential to integrate mental health care into routine HIV care to improve treatment outcomes [32–34]. Routine HRQoL and CMDs assessments using generic and condition-specific outcomes followed by appropriate treatment plans incorporating the multi-disciplinary team approach are paramount [35].

We found individuals in relationships or married to have HRQoL higher than those divorced, widowed, or not in a relationship. Marriage or companionship is an essential source of social support. Social support is an essential buffer to stressful life events; it positively impacts an individual’s physical and mental well-being, performance, creativity and competence and brings resilience when confronting stressful situations [36–38]. For example, an Ethiopian systematic review showed that PLHIV with good social support were four times more likely to report higher HRQoL when compared to those without (AOR = 4.01, 95% CI 3.07–5.23) [39]. Our findings further affirm previous evidence on the intertwined relationship between social support, adherence to ART and improvement of the overall HRQoL for PLHIV [40–43].

Consistent with a previous meta-analysis [44], participants who had lived longer with HIV reported a higher HRQoL. Those recently diagnosed may have been in the bereavement process and had not yet accepted their HIV status, leading to poor mental health functioning [45]. More extended periods of awareness and ART intake can facilitate the growth of effective coping approaches, leading to improved mental health and higher HRQoL [44]. In this study, adhering to ART was predictive of improved HRQoL. Increased ART coverage is associated with reduced morbidity, improved immune functioning and psychological well-being, ultimately increasing the overall perceived HRQoL [44]. Also, HIV care centres in Zimbabwe offer infected individuals free counselling and peer support groups [46]. People with more extended treatment periods may have been subjected to formal and informal psychosocial support, which may have led to better acceptance and adherence to care, thus reducing morbidity and improving HRQoL overall [15, 16, 35, 47].

In this study, financial inadequacy and food insecurity, proxy indicators for financial well-being, were predictive of poor HRQoL. For example, participants with adequate finances were four times more likely to have better HRQoL than those with inadequate finances. Our findings follow a previous systemic review showing the link between HRQoL and financial well-being (R^2^ = 0.108, p = .04) [11]. The relationship between food security and HIV infection is complex and bidirectional. For instance, food insecurity and HIV infection result in a gradual decline of immunity which may lead to a lower HRQoL [48–50]. Further, most Zimbabwean PLHIV incur out-of-pocket medical expenses, shrinking their incomes; the financial pressure negatively affects HRQoL. Therefore, providing social and economic incentives for low-income PLHIV should be considered to improve their HRQoL [51].

Further, the experience of adverse events negatively impacted the HRQoL in PLHIV. Commonly reported adverse events included failing a course, bereavement, and loss of income-generation opportunities. Experiencing an adverse event negatively affects mental and physical health as patients may fear reliving past traumatic experiences [25, 26, 44]. Consequently, the burden may lead to troubling memories, poor mental health functioning, and a lower HRQoL [52, 53]. Last, higher education attainment was associated with improved HRQoL; this is consistent with previous systematic reviews [11, 54]. A higher education facilitates stronger awareness of the disease and a better ability to cope with the challenges of a chronic illness [11, 54–57]. Conversely, lower education, a proxy indicator of poor financial well-being [56], is associated with poor health-seeking behaviours; this further reinforces the need for social and financial support for poorer PLHIV and their families.

## Conclusion

Overall, our study findings show high self-evaluation of HRQoL among Zimbabwean PLHIV. Being aware of HIV status for over a year, not experiencing an adverse event, being currently married, having adequate finances and food security and having higher educational status were associated with higher HRQoL. Also, CMDs were highly prevalent. Consequently, we recommend continually integrating mental health services into routine HIV care. Also, periodic HRQoL evaluation to inform evidence-based care is warranted. Last, there is a need to develop and implement bespoke multisectoral interventions to improve the HRQoL of Zimbabwean PLHIV.

### Limitations

There are some limitations to our study. First, applying a facility-based, cross-sectional study may have introduced selection bias, and causality cannot be inferred. Further prospective studies recruiting community indwelling patients are needed to understand the longitudinal changes in HRQoL over time fully. Also, we selected participants non-randomly, which may have introduced selection bias. However, a strength of this study was the recruitment of a large sample and using locally-validated, internal HRQoL measures.

### Electronic supplementary material

Below is the link to the electronic supplementary material.

Supplementary Material 1

## Data Availability

The datasets used and/or analysed during the current study are available from the corresponding author on reasonable request. The datasets will be availed onto online repositories once all manuscripts related to the study have been published online.

## List of abbreviations

PLHIV: Persons living with HIV
ART: Antiretroviral Therapy
EQ5D-5L: The 5 level EuroQOL 5 Dimensions
HAT-QOL: HIV/AIDS - Targeted Quality of Life
HIV: Human Immune-deficiency Virus
HRQOL: Health Related Quality of Life
PLWHIV: People living with HIV
UNAIDS: The Joint United Nations Program on HIV/AIDS
WHOQOL-HIV-BREF: World Health Organization Quality of Life Human Immuno-Deficiency Virus BREF
CMD: Common Mental Disorder
