# Supplementary figures and images for: Evaluation of the health-related quality of life and associated factors in Zimbabwean adults living with HIV: a cross-sectional study
Source: BMC Res Notes. 2023 Oct 4;16:251. doi: 10.1186/s13104-023-06536-3 (PMC10548739; doi:10.1186/s13104-023-06536-3)

**Supplementary File 1: Socio-demographic Questionnaire**

| 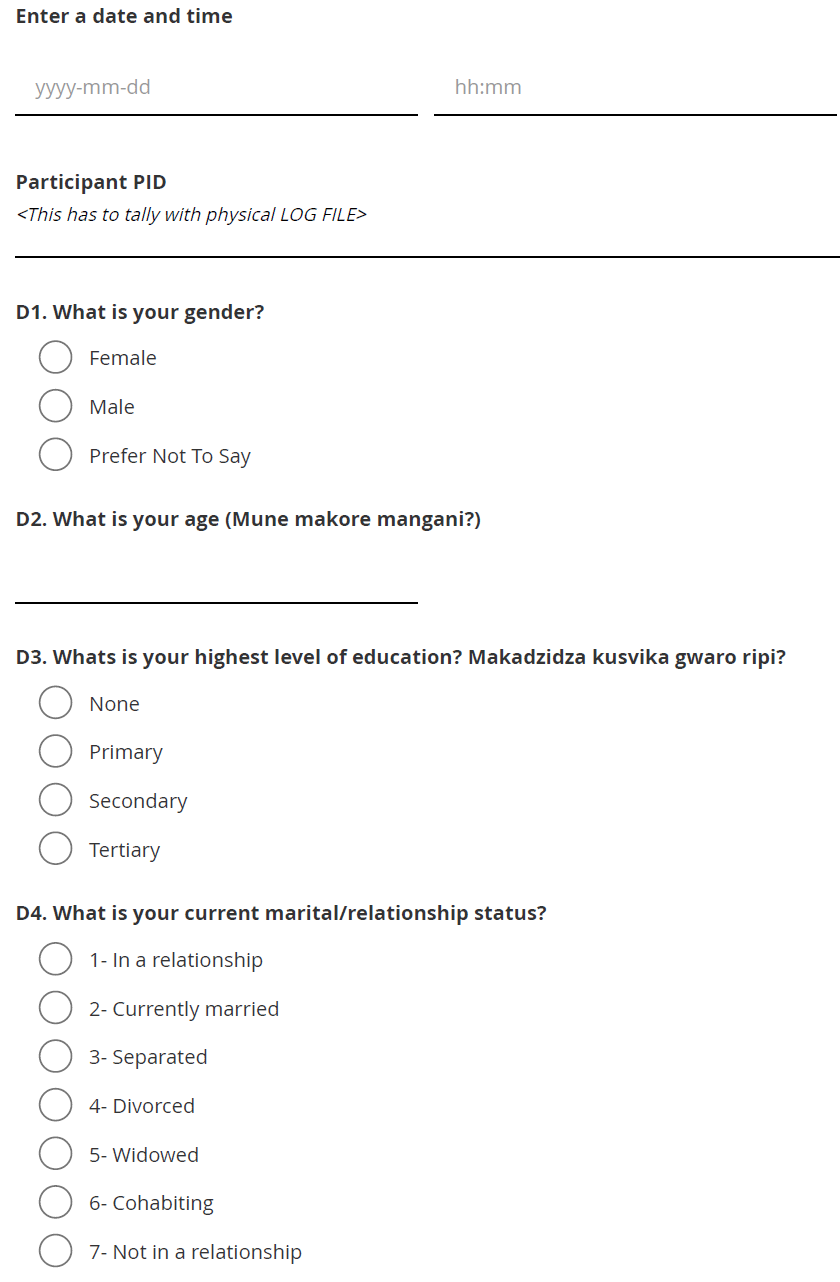 | 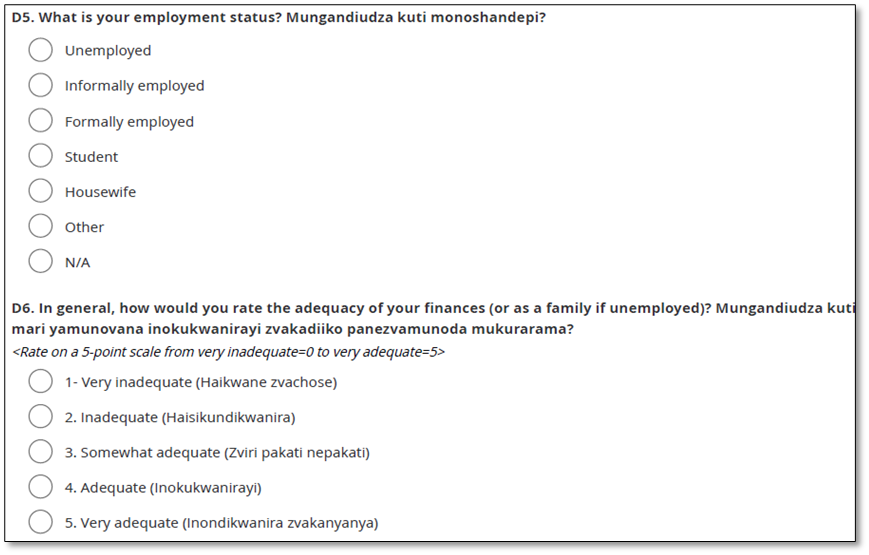 |
| --- | --- |
| 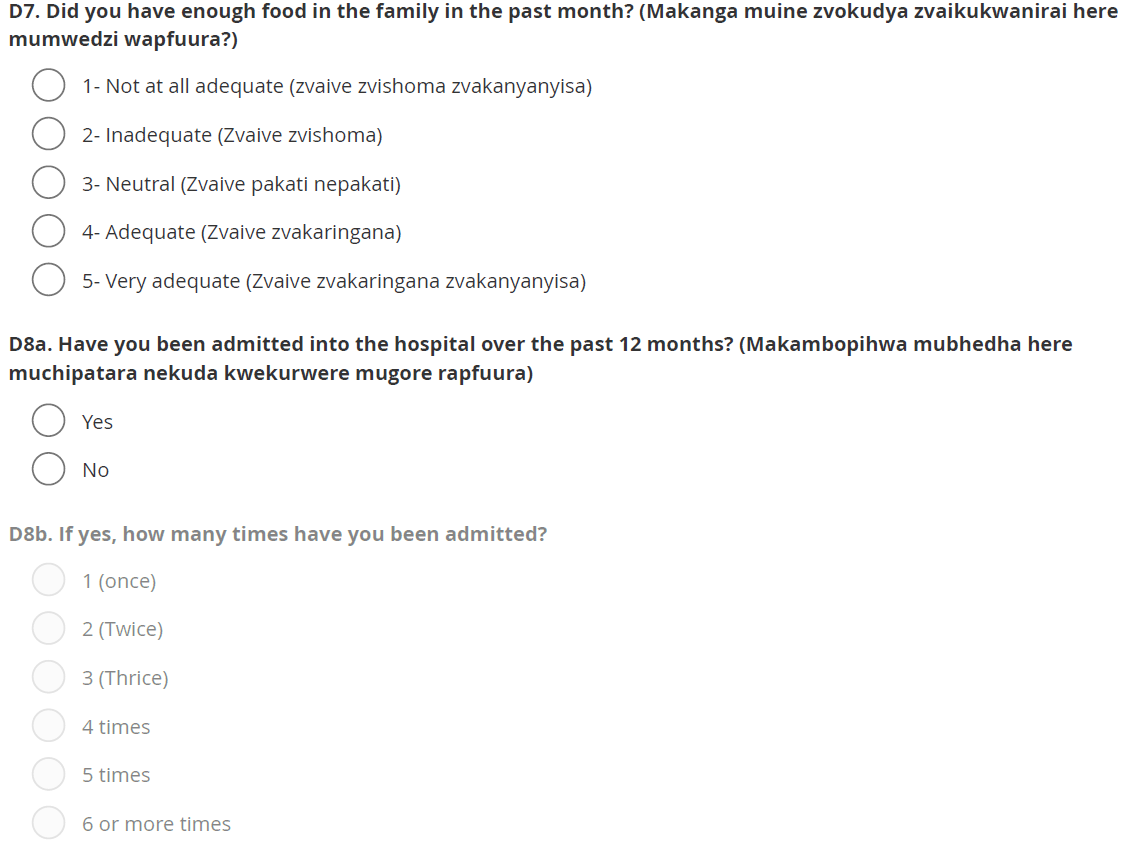 | |


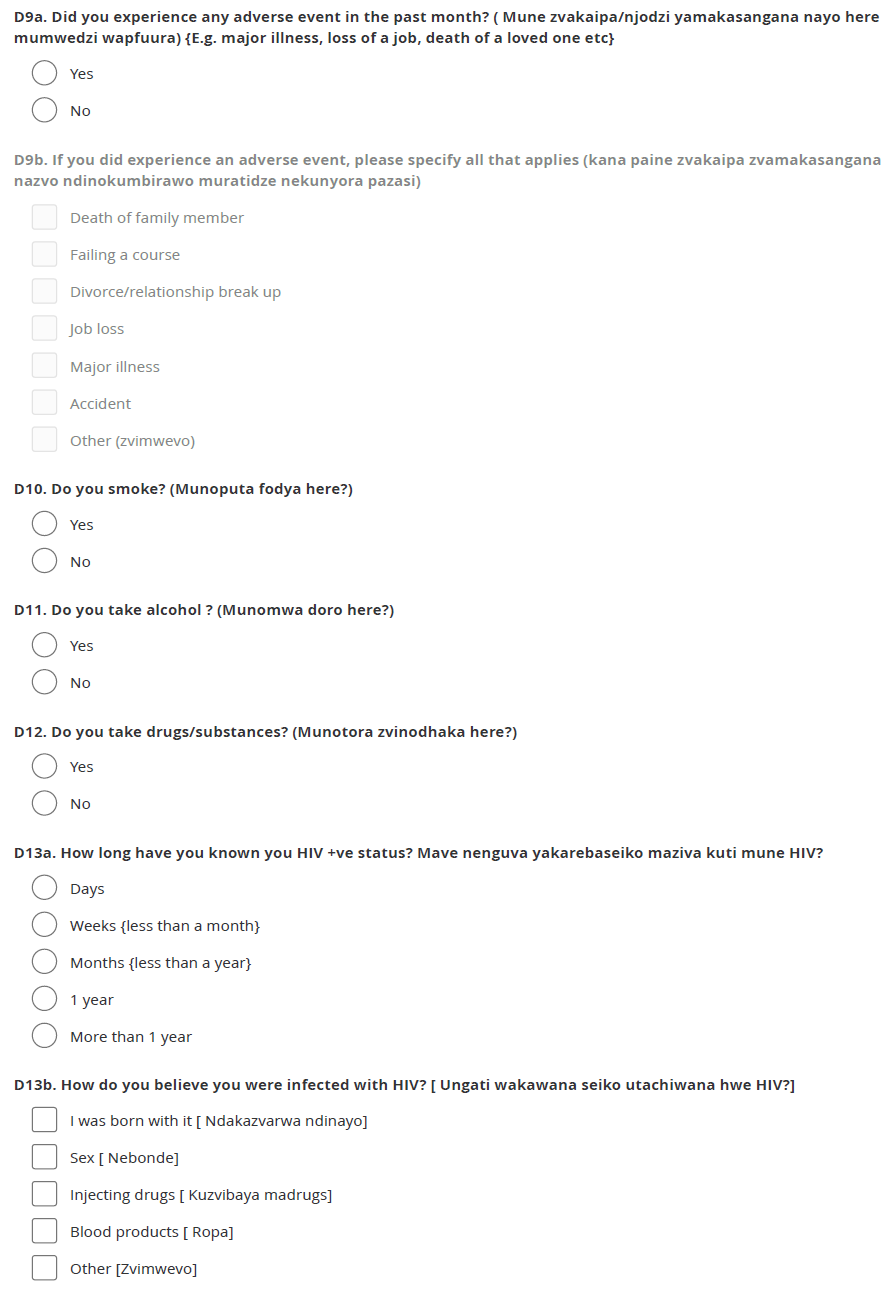


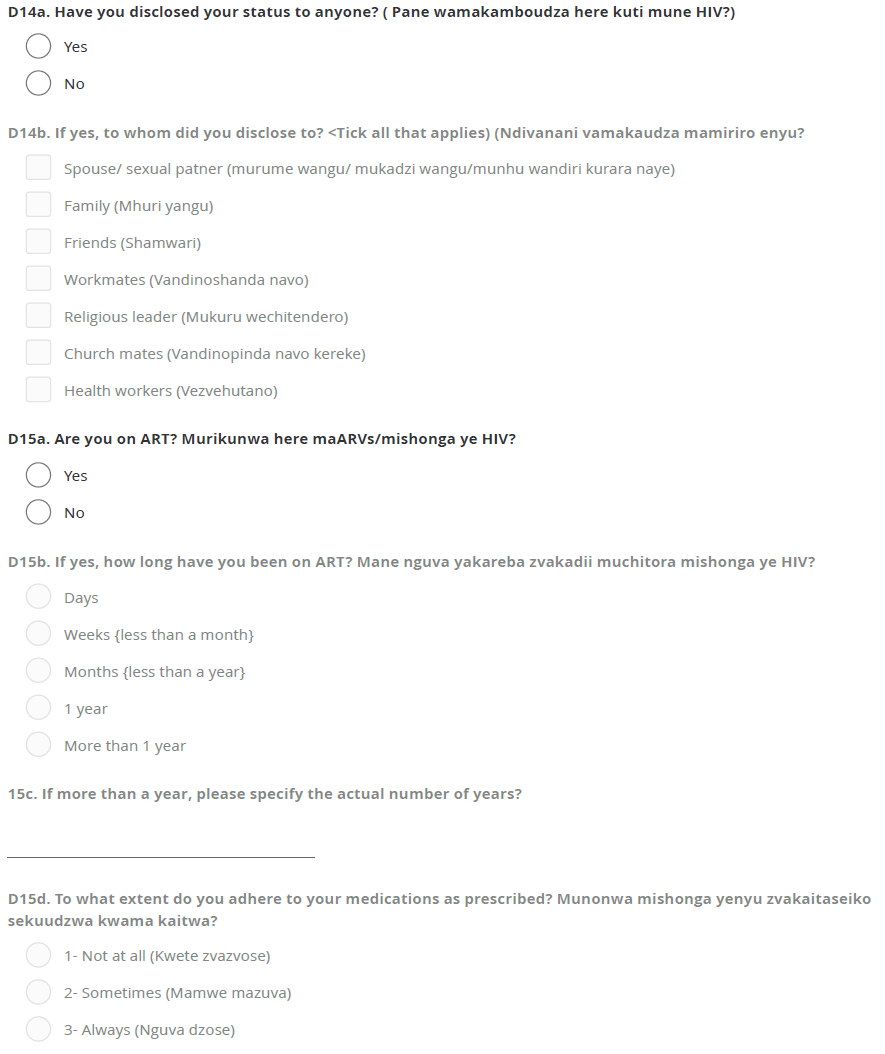

Supplement: Supplementary file 1 — Supplementary Material 1 [file 13104_2023_6536_MOESM1_ESM.docx]
